# Physician engagement in reproductive health advocacy: findings from a mixed methods evaluation of a leadership and advocacy program

**DOI:** 10.1186/s12909-024-05410-5

**Published:** 2024-04-30

**Authors:** Heidi E. Jones, Meredith Manze, Anita Brakman, Amy Kwan, MiQuel Davies, Diana Romero

**Affiliations:** 1 City University of New York Graduate School of Public Health and Health Policy (CUNY SPH), 55 W. 125th St #7th Floor, 10027 New York, NY USA; 2 City University of New York Institute of Implementation Science in Population Health (CUNY ISPH), New York, USA; 3 Physicians for Reproductive Health, Hartsdale, NY USA

**Keywords:** Physician training, Advocacy, Reproductive health, Abortion

## Abstract

**Background:**

Medical curricula include advocacy competencies, but how much physicians engage in advocacy and what enables this engagement is not well characterized. The authors assessed facilitators and barriers to advocacy identified by physician alumni of a reproductive health advocacy training program.

**Methods:**

The authors present secondary results from a mixed methods program evaluation from 2018 to 2020, using alumni data from a cross-sectional survey (*n* = 231) and in-depth interviews (IDIs, *n* = 36). The survey measured engagement in policy, media, professional organization, and medical education advocacy and the value placed on the community fostered by the program (eight questions, Cronbach’s alpha = 0.81). The authors estimated the association of community value score with advocacy engagement using multivariable Poisson regression to estimate prevalence ratios and analyzed IDI data inductively.

**Results:**

Over one third of alumni were highly engaged in legislative policy (*n* = 90, 39%), professional organizations (*n* = 98, 42%), or medical education (*n* = 89, 39%), with fewer highly active in media-based advocacy (*n* = 54, 23%) in the year prior to the survey. Survey and IDI data demonstrated that passion, sense of urgency, confidence in skills, and the program’s emphasis on different forms of advocacy facilitated engagement in advocacy, while insufficient time, safety concerns, and sense of effort redundancies were barriers. The program community was also an important facilitator, especially for “out loud” efforts and for those working in environments perceived as hostile to abortion care (e.g., alumni in hostile environments with high community value scores were 1.8 times [95% CI 1.3, 2.6] as likely to report medium/high levels of media advocacy compared to those with low scores after adjusting for age, gender, and clinical specialty).

**Conclusion:**

Physician advocacy training curricula should include both skills- and community-building and identify a full range of forms of advocacy. Community-building is especially important for physician advocacy for reproductive health services such as abortion care.

## Background

Physicians play an important role in advocating for policies and systems that promote their patients’ health [1]. Advocacy is included as a core curriculum requirement by the Accreditation Council for Graduate Medical Education [2]. Yet, in a 2017 survey of Family Medicine Residency Program Directors in the United States (US), only one-third reported requiring advocacy in their curriculum [3].

Post-residency advocacy training programs can supplement residency programs to hone physicians’ advocacy skills and increase their level of advocacy engagement. One such program is Physicians for Reproductive Health’s Leadership Training Academy (LTA), a 9-month physician training to develop leadership in advocacy on sexual and reproductive health, including abortion and contraceptive care, in the US. The training program started in 2004 for post-residency family planning fellows, before broadening to physicians outside the fellowship in 2012. Additionally, the training program began with small cohorts of less than 5 students from 2004 to 2009 (no new students in 2005) to larger cohorts of 20 to 30 students from 2010 onward. The curriculum focuses on skills related to communication, media, interacting with policymakers, and leadership, in instructional workshops that last three to five days every three months, with on-line webinars and one-on-one feedback interspersed. Program applicants are selected based on professional aspirations and goals, leadership experience and potential, commitment to LTA values/goals, policy/media readiness, and regional and lived experience diversity.

While a 2004 survey found that over 90% of US physicians rated advocacy as important to their profession, at least one-third had not engaged in any advocacy work in three years prior to the survey [4]. Similarly, in 2018 a survey of 886 physicians at the University of Wisconsin, found 21% reported not being engaged in any type of health policy or advocacy activities. As restrictions to reproductive health, especially abortion [5, 6], increase in the US, the role of physicians in advocating for patients’ access to these services is critical. Identifying barriers and facilitators to physician engagement in advocacy can inform training curricula. We present secondary results from a mixed methods evaluation of the LTA program from 2018 to 2020 to describe physicians’ post-training advocacy engagement and its barriers and facilitators.

## Methods

The mixed methods evaluation included a survey and in-depth interviews (IDIs) of alumni (as of 2018). The survey asked questions on advocacy efforts across four domains targeted by the curriculum—legislative policy (e.g., interacting with policymakers), media (e.g., interviews with television/radio), professional organization (e.g., participation in committees), and medical education (e.g., lecturing on abortion service delivery); the value they place on the LTA program community; and sociodemographic characteristics.

We sent the on-line survey to all alumni from 2004 to 2018 (*n* = 326) by email with reminders from September to November 2018. In total, 231 (71%) completed 80% or more of the survey, with no significant differences by response for age, race, ethnicity, gender, clinical specialty, or participation in post-residency fellowship; we therefore present unweighted data. The first page of the survey included informed consent language with survey entry indicating consent. The survey included 97 questions altogether with 31 min as the median time of completion. Participants received $35 giftcards.

We categorized engagement as low, medium, or high in each domain based on types of activities reported in the last year (one or no activity coded as low, two or three as medium, and four or more as high). For legislative policy, we incorporated frequency of policymaker meetings; those who met with policymakers almost every week were coded as high, and once or twice a month as medium, regardless of number of types of activities. Similarly, for media, those who reported posting on social media almost every day in combination with at least one other form of media advocacy were coded as high; those who posted on social media almost every day or once a week as their only form of media advocacy were coded as medium.

The IDI guide development included questions on advocacy activities, program community engagement, and barriers and facilitators to advocacy. Survey results informed probes to operationalize categories of engagement and barriers and facilitators to advocacy engagement. Further, we used purposive sampling based on survey results to identify a subgroup of alumni (*n* = 44), with variation in low, medium, and high levels of advocacy across the domains; self-reported level of hostility toward abortion in their work environment; years of practicing medicine; or not having responded to survey (*n* = 6); 36 (82%) completed IDIs. Participants provided informed consent and received $50 giftcards. We used iterative inductive analysis informed by grounded theory methodology. Three authors [AK, MM, DR] double-coded a subset of transcripts, revised codes to increase analytic dependability, and created a detailed code structure. The current analysis focuses on themes related to engagement in advocacy. Perspectives on what constitutes effective advocacy are reported elsewhere [7]. Pseudonyms are used for illustrative quotes, with overall engagement level indicated in parentheses.

Using survey data, we present descriptive statistics on levels of engagement and types of advocacy activities. We estimated a Spearman’s correlation to test the extent to which level of engagement in advocacy domains were correlated. We combined eight 5-point Likert questions on the value of the LTA program community into a score (Cronbach’s alpha = 0.81). Results from the IDIs informed our analytic approach to the survey. Given that the importance of the program community emerged in the IDIs, for the survey we tested whether community value (dichotomized at the median, given generally high scores) was associated with engagement using Poisson regression with robust standard errors to estimate prevalence ratios, after adjusting for age, gender, and clinical specialty as *a priori* hypothesized confounders. Similarly, qualitative data suggested that clinicians working in regions with low levels of support for abortion care, relied on social support provided by the program community more heavily than those working in highly supportive environments. We therefore stratified this analysis by self-reported local level of hostility to abortion (very/somewhat versus little/no hostility) to test whether this association differed by level of hostility and present p-values for interaction when stratum specific estimates differed. We also explored whether level of engagement in each of the advocacy domains was associated with age, gender, clinical specialty, alumni cohort (2004–2009, 2010–2014, 2015–2018) and participation in post-residency family planning fellowships using Chi-Squared or Fisher’s exact tests, as appropriate. We compared survey results with thematic findings. We present integrated quantitative and qualitative findings by advocacy domain, facilitators and barriers to advocacy and the role of community.

Researchers from the City University of New York Graduate School of Public Health and Health Policy led data collection and analysis; two program authors [AB, MD] were blinded to individual-level data. The study was approved by the CUNY SPH’s Institutional Review Board (protocol 2018 − 1045).

## Results

The median age of respondents was 38 years (range 31 to 79); 58% had completed the program in 2015–2018, 36% in 2010–2014 and 6% in 2004–2009. The majority identified as women (*n* = 211, 91%), with 65% (*n* = 151) identifying as white, 15% (*n* = 34) Asian/Pacific Islander, 7% (*n* = 16) Black, 5% (*n* = 12) Hispanic, and 8% (*n* = 18) mixed race/other. The most represented clinical specialties were obstetrics and gynecology (*n* = 156, 68%) and family medicine (*n* = 59, 26%). Almost all (*n* = 226, 98%) were practicing medicine at the time of the survey and 87% (*n* = 200) had provided abortions in the previous year. Most alumni (*n* = 183, 79%) practiced medicine in urban settings, with all but six states represented. For the level of hostility toward abortion in the area where they do most of their clinical care, 16% (*n* = 37) reported very hostile, 21% (*n* = 48) somewhat hostile, 34% (*n* = 77) a little hostile and 28% (*n* = 63) not hostile environments (*n* = 6, 3% were not practicing or did not respond to this question).

### Overall advocacy

Over one-third of alumni were highly engaged in professional organization advocacy work, medical education, or legislative policy advocacy, with fewer highly active in media-based advocacy (Table 1). Only three (1%) were highly active across all domains, and three (1%) not active across all domains. Those who reported being engaged in legislative policy were more likely to be engaged with media (Spearman’s ρ = 0.46), and those who reported being active in professional organizations were more likely to be engaged in medical education (Spearman’s ρ = 0.38). Findings from the IDIs suggested that the program’s emphasis on different forms of advocacy facilitated physicians’ ability to leverage their strengths.

…if you think that whoever has time to be on MSNBC has time to sit on three committee meetings a week at a hospital to get something done, that’s a fallacy, that can’t be the same person doing all of that. The wonderful part of the LTA is it gives you potential lanes and the skills of what you do in that lane.– Sandy [low].

**Table 1:** Self-reported levels of engagement in past year across four advocacy domains by clinical specialty, importance of advocacy training program community, and level of hostility toward abortion in the area; Mixed methods evaluation of physician advocacy training program, 2018–2020

Level of advocacy engagement	N (%)	Clinical specialty, n (%)				Value of training program community score, n (%)*			Level of hostility to abortion in work area, n (%)*		
	Total	Obstetrics/ Gynecology (*n* = 156)	Family Medicine (*n* = 59)	Other (*n* = 16)	p-value	Higher (above median) (*n* = 123)	Lower (below median) (*n* = 104)	p-value	Very/ somewhat (*n* = 85)	Little/ not (*n* = 140)	p-value
Legislative Policy											
Low	45 (19.5)	34 (21.8)	10 (16.9)	1 (6.3)	0.27	16 (13.0)	29 (27.9)	0.02	7 (8.2)	37 (26.4)	< 0.01
Medium	96 (41.5)	58 (37.2)	28 (47.5)	10 (62.5)		54 (43.9)	41 (39.4)		39 (45.9)	55 (39.3)	
High	90 (39.0)	64 (41.0)	21 (35.6)	5 (31.3)		53 (43.1)	34 (32.7)		39 (45.9)	48 (34.3)	
Media*											
Low	83 (36.6)	53 (34.9)	27 (45.8)	3 (18.8)	0.31	38 (31.9)	45 (43.3)	0.19	28 (34.1)	53 (38.1)	0.08
Medium	90 (39.6)	61 (40.1)	21 (35.6)	8 (50.0)		50 (42.0)	39 (37.6)		28 (34.1)	60 (43.2)	
High	54 (23.8)	38 (25.0)	11 (18.6)	5 (31.3)		31 (26.1)	20 (22.9)		26 (31.7)	26 (18.7)	
Professional Organization*											
Low	49 (21.3)	31 (20.0)	11 (18.6)	7 (43.8)	0.33	22 (17.9)	27 (26.2)	0.17	16 (19.0)	30 (21.4)	0.31
Medium	83 (36.1)	56 (36.1)	23 (39.0)	4 (25.0)		51 (41.5)	32 (31.1)		27 (32.1)	56 (40.0)	
High	98 (42.6)	68 (43.9)	25 (42.4)	5 (31.3)		50 (40.7)	44 (42.7)		41 (48.8)	54 (38.6)	
Medical Education*											
Low	40 (17.6)	20 (13.0)	17 (29.3)	3 (20.0)	0.07	17 (14.0)	22 (21.6)	0.22	12 (14.3)	26 (19.0)	0.28
Medium	98 (43.2)	72 (46.8)	19 (32.8)	7 (46.7)		58 (47.9)	39 (38.2)		33 (39.3)	62 (45.3)	
High	89 (39.2)	62 (40.3)	22 (37.9)	5 (33.3)		46 (38.0)	41 (40.2)		39 (46.4)	49 (35.8)	

* six cases missing response on level of hostility, four missing media, four missing medical education, four missing advocacy training program community score, and one missing professional organization

### Legislative policy advocacy

In the year prior to the survey, 77% (*n* = 178) reported emailing, writing, or calling policymakers, 49% (*n* = 113) met with policymakers in person, and 32% (*n* = 75) provided context expertise to policymakers. Legislative policy advocacy was associated with higher community value scores (Table 1) with no differences by age, gender, clinical specialty, time since graduation, or participation in post-residency fellowship. Those working in hostile environments to abortion were more likely to report interacting with policymakers frequently; 36% (*n* = 31/85) of those in very/somewhat hostile environments reported interacting with policymakers every week compared to 16% (*n* = 22/140) of those in little/not hostile environments (*p* < 0.01).

In the IDIs, many alumni attributed the program with preparation needed to engage in policy-related advocacy and highlighted the importance of the LTA network.

I feel it [policy-related advocacy activity] was non-existent before the LTA. That I didn’t really know how to approach reaching out to my local politicians, or my national politicians about these issues. I feel after LTA, I had much more of a support system and just a better understanding of how to do that, and so felt much more confident reaching out to those people to make them aware of certain reproductive health issues.– Rafaela [high].

### Media advocacy

In terms of media activities, about one-quarter of alumni survey respondents (24%) were highly engaged, 40% reported medium levels of engagement and 37% low levels (Table 1). In the last year, 54% (*n* = 123) had interviewed with print media; 38% (*n* = 86) wrote an opinion piece/commentary; and 29% (*n* = 67) wrote a letter to the editor. One-quarter (25%, *n* = 57) had participated in telephone interviews and 23% (*n* = 52) were interviewed on camera. Level of engagement in media was not associated with age, gender, clinical specialty, time since graduating or participation in post-residency fellowship.

### Professional organization advocacy

The most common types of professional organization advocacy reported were participating in a committee, working group or task force (*n* = 149, 65%), developing organizational/institutional policies (*n* = 144, 63%), or attending professional organization advocacy events (*n* = 115, 50%). The level of engagement was not associated with age, clinical specialty, time since graduation or post-residency fellowship. However, men were more likely than women to engage in professional organization activities; 58% (*n* = 11/19) of men versus 41% (*n* = 86/210) of women reported high levels of this type of engagement (*p* < 0.01).

A common theme in the IDIs centered on increased awareness, from the program, of the importance of participating in professional medical societies and the incremental nature of this type of work (“it’s a marathon not a sprint”).

I sit on a committee for the [state’s group for family physicians], which is an opportunity that I wouldn’t necessarily have gotten involved in. We don’t do anything specific to reproductive health now, but again, a lot of it is the long game of getting my foot in the door at [this professional organization], so that I can make a long-term difference. Just even having that on my radar as something to do, would not have come out without PRH and the LTA.– Diana [medium].

### Medical education advocacy

The most common forms of medical education reported were teaching/lecturing students (*n* = 194, 84%), developing educational material/curriculum (*n* = 127, 55%), and presenting at a panel/conference (*n* = 124, 54%). Medical education efforts were common among those who reported participating in post-residency fellowships in academic medicine—89% (*n* = 136/152) of those in a fellowship had medium/high levels of engagement versus 68% (*n* = 51/75) of those not in a fellowship (*p* < 0.01).

### Barriers/Facilitators

**Fig. 1:**
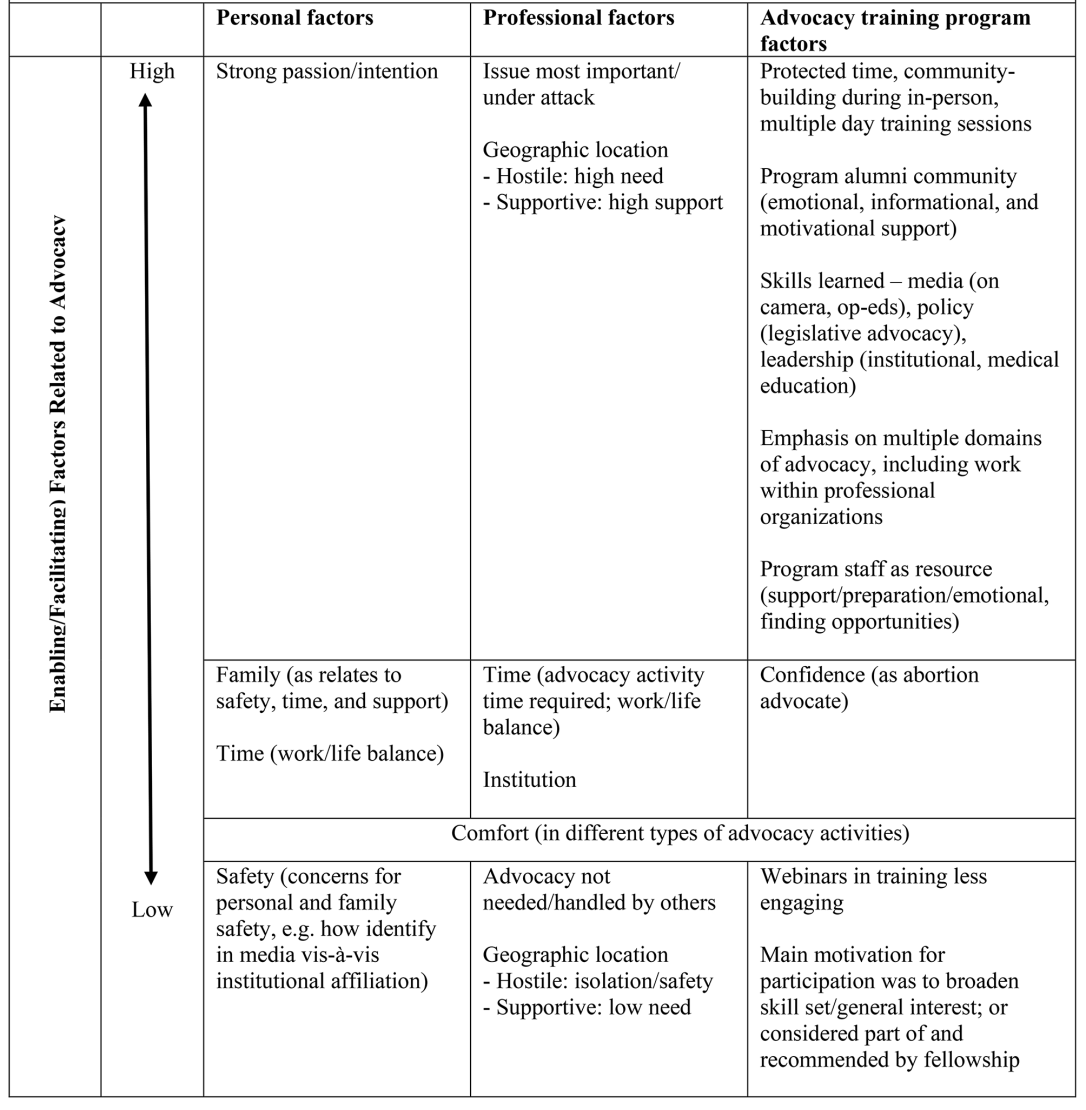
Relative importance of factors that facilitate physician’s engagement in reproductive health-related advocacy, especially abortion; Mixed methods evaluation of physician advocacy program, 2018–2020

Factors influencing engagement identified in the IDIs were categorized into personal, professional, and program-related (Fig. 1). Factors related to high levels of engagement included: passion regarding reproductive health (*personal*); urgency of issue (*professional*); skills fostered by the program (*program-related*). Factors related to low levels of engagement included: concerns regarding personal/family safety especially for ‘out loud’ activities (*personal*); advocacy handled by others (*professional*); and less successful aspects of the training (*program-related*).

Many of these factors were confirmed in survey responses. When provided with a list of abortion advocacy facilitators, the top five selected were: motivation to promote social or reproductive justice (*n* = 177, 77%); motivation to increase abortion care access (*n* = 170, 74%); supportive network of LTA colleagues (*n* = 146, 63%); supportive family/friends (*n* = 140, 61%) and sense of important/valuable contribution (*n* = 131, 57%). The number one barrier to abortion advocacy reported was lack of time (*n* = 160, 69%), followed by concerns for safety of family (*n* = 74, 32%) and concerns about being harassed (*n* = 69, 30%).

Confidence and comfort in abortion advocacy were situated in the center of the engagement continuum. In both the survey and IDIs, alumni generally reported *confidence* in their role as abortion advocates—96% (*n* = 222) of survey respondents reported that the program increased their confidence as abortion advocates. However, alumni described their *comfort* with advocacy as situational, varying by topic, setting/audience, and communication modality.

### Role of community

Generally, alumni felt positively about the LTA community (Table 2). After adjusting for clinical specialty, age, and gender, highly valuing the LTA community was modestly associated with reporting medium/high levels of engagement in advocacy. Valuing community was especially important for alumni reporting working in areas hostile to abortion for public-facing media efforts (test for interaction *p* < 0.01, Table 3).

**Table 2:** Alumni reports on feelings about the LTA program community and community score; Mixed methods evaluation of physician advocacy program, 2018–2020

Statements rated from strongly agree (1) to strongly disagree (5)	Total (*n* = 227)*
Positive statements: Strongly agree/agree, n (%)	
The LTA community has had a major influence on abortion care in the U.S.	198 (86.1)
The LTA community does not give up during tough times	226 (98.3)
Members of the LTA community look out for each other	201 (87.8)
The support of the people I met through the LTA help me to be an active abortion advocate	194 (84.3)
Being part of the LTA makes me feel less isolated as an abortion advocate	208 (90.4)
I am frequently in touch with people met through the LTA	138 (60.0)
Negative statements: Strongly disagree/disagree, n (%)	
I’m unhappy with the LTA community’s level of commitment to our goals	208 (90.8)
Members of the LTA community do not connect with one another	182 (79.1)
Mean LTA Community Value Score (SD)** (Range 1–5, with 1 = strongly agreeing with positive community attitudes)	1.6 (0.6)

*four cases missing response to one or more of these measures; **summary score after reverse coding responses to negative statements

**Table 3:** Relationship between strong sense of community among program alumni and medium/high level of engagement by advocacy domain and by level of hostility of work environment to abortion, Mixed methods evaluation of physician advocacy training program, 2018–2020

Above median LTA community score as a predictor of high/medium levels of engagement in:	Somewhat/very hostile environment to abortion (*n* = 85) aPR (95% CI)*	Little/not hostile environment to abortion (*n* = 140) aPR (95% CI)*	Total (*n* = 223–227)** aPR (95% CI)*
Legislative policy advocacy	1.2 (1.0, 1.4)	1.3 (1.0, 1.6)	1.2 (1.1, 1.4)
Media advocacy	1.8 (1.3, 2.6)	1.0 (0.8, 1.3)	1.2 (1.0, 1.5)
Professional organization advocacy	1.1 (0.9, 1.4)	1.2 (1.0, 1.4)	1.1 (1.0, 1.2)
Medical education advocacy	1.1 (0.9, 1.3)	1.1 (0.9, 1.4)	1.1 (1.0, 1.3)

*adjusted prevalence ratio (aPR), adjusted for clinical specialty, age, and gender

** sample size varies based on missing responses for each advocacy domain

The value of the LTA community as a facilitator to advocacy engagement emerged from the IDIs as well. For many, the LTA community functioned as a source of informational, motivational, and emotional support. As Lauren [medium] said, “you feel there’s no one else out there that cares about these issues, so it was very nice to be able to find your people.” Patti [high] expanded on this:

… it was a safe space inside a community of reproductive health providers… And I think that some of the stigma associated with being a provider for abortion or contraception care means that leadership looks different, or has to look different, or it’s more important to have very highly evolved leadership skills in order to overcome that stigma and also be strategic around the advocacy in that area. And I think that having that protected space inside that community is extremely important.

Further, as Robert [medium] stated, “part of me continuing to be an abortion provider is feeling responsibility to the other members of the LTA class that I was in, as well as my own personal motivations…creating that shared responsibility…was really powerful.” Sally [high] described the importance of community for informational support:

I think that for those of us who have gone through the LTA, who continue to be pretty active in advocacy, particularly the abortion advocacy and in the not-so-comfortable spaces…I will say that we have a really great relationship between all of us, where we can… shoot ideas back and forth, practice questions with each other, do review talking points. I think that we rely a lot on each other in that space, both for the content, the knowledge, the practice, and also for just like, “Okay, it’s going to be fine.”

## Discussion

In this mixed methods evaluation of the LTA advocacy and leadership training program, survey and in-depth interview findings informed one another to gain a comprehensive understanding of barriers and facilitators to advocacy engagement. We found moderate to high levels of engagement of advocacy among most alumni, with alumni often focusing efforts on one or two domains. Physician engagement in legislative policy was correlated with engagement with the media, while engagement in professional organizations was correlated with engagement in medical education. Both legislative policy and media advocacy efforts are public facing, while work within professional organizations and medical education involve interactions with other physicians, which may explain this grouping of effort; alumni likely aligned advocacy efforts within their comfort zone. One of the strengths of the training program identified by alumni was that it taught them to consider which advocacy domain(s) to focus on based on their strengths, skills, and personal and professional considerations.

In general, fewer alumni were involved in media advocacy than in other domains. This may be due to ‘out loud’ abortion advocacy in the media being associated with safety risks in the US. In a study of 88 individuals who shared their abortion stories publicly as part of two abortion story-sharing campaigns, 60% reported experiencing harassment after this publicity, with 14% reporting receiving death threats [8].

We found that men were more likely than women to report engagement with medical professional organization efforts. Historically, in the US, women have had less access to leadership roles in medicine than men. A study of medical school graduates from 1979 to 2013 found that women physicians at academic medical centers were less likely than men to be promoted to higher faculty ranks or to be made department chairs, and this difference did not wane over time [9]. Training programs, such as the LTA may help rectify this situation, as most program participants identified as women, and many reported not having considered participation in professional organizations and/or seen the relevance for advocacy in this domain prior to the program.

We found that moderate and high levels of engagement in advocacy, especially public-facing advocacy, were associated with the strength of value placed on the community of colleagues established by the training program. This sense of community was especially important for media engagement (i.e. ‘out loud’ advocacy) for physicians working in areas they perceived as hostile to abortion care. Previous research has shown the importance of interpersonal communication among physicians in reducing burnout and compassion fatigue among abortion providers [10]. Advocacy efforts, especially public-facing efforts, may equally require support from physician colleagues for sustainability. Advocacy training curricula should include community-building in addition to skills-building.

These findings should be interpreted within the limitations of our study. We created the low, medium, and high advocacy engagement levels as we could not find previously validated measures; however, triangulation of findings with IDIs increased confidence in the validity of these categories. All data are self-reported; social desirability may have impacted physicians’ reports.

## Conclusion

Advocacy is an important component in medical education training. Programs such as the LTA may increase physicians’ confidence to engage in advocacy and to identify the advocacy domains that speak to their strengths and professional and personal circumstances. Establishing a community of colleagues is an important element of physician advocacy training programs to ensure sustainability of advocacy. This need for physician community-building may be especially important in the US within the realm of abortion services, which continue to be subject to increased restrictions [6], especially in the context of the Supreme Court’s Dobbs decision [5]. Given such continued restrictions, increasing advocacy training in medical and post-medical curricula can help to broaden advocacy efforts to ensure equitable access to abortion.

## Data Availability

The datasets generated and/or analysed during the current study are not publicly available due to the sensitive nature of identifying providers of abortion and contraception in the US but are available from the corresponding author on reasonable request.

## Abbreviations

aPR: adjusted prevalence ratio
CI: confidence interval
IDIs: in-depth interviews
LTA: Leadership Training Academy
PRH: Physicians for Reproductive Health
US: United States
